# Tailored Algorithm for Sensitivity Enhancement of Gas Concentration Sensors Based on Tunable Laser Absorption Spectroscopy

**DOI:** 10.3390/s18061808

**Published:** 2018-06-04

**Authors:** Everardo Vargas-Rodriguez, Ana Dinora Guzman-Chavez, Roberto Baeza-Serrato

**Affiliations:** Departamento de Estudios Multidisciplinarios, División de Ingenierías, Universidad de Guanajuato, Av. Universidad s/n, Col. Yacatitas, Yuriria, Gto C.P. 38940, Mexico; r.baeza@ugto.mx

**Keywords:** gas sensors, optical sensors, optoelectronic and photonic sensors, signal processing in optics

## Abstract

In this work, a novel tailored algorithm to enhance the overall sensitivity of gas concentration sensors based on the Direct Absorption Tunable Laser Absorption Spectroscopy (DA-ATLAS) method is presented. By using this algorithm, the sensor sensitivity can be custom-designed to be quasi constant over a much larger dynamic range compared with that obtained by typical methods based on a single statistics feature of the sensor signal output (peak amplitude, area under the curve, mean or RMS). Additionally, it is shown that with our algorithm, an optimal function can be tailored to get a quasi linear relationship between the concentration and some specific statistics features over a wider dynamic range. In order to test the viability of our algorithm, a basic C${}_{2}$H${}_{2}$ sensor based on DA-ATLAS was implemented, and its experimental measurements support the simulated results provided by our algorithm.

## 1. Introduction

Gas sensors based on Tunable Laser Absorption Spectroscopy (TLAS) are widely used due to their high sensitivity and selectivity. Moreover, there currently exists a wide range of laser options to implement this application. Generally, TLAS can be performed in two different modes: Line Locked Tunable Laser Absorption Spectroscopy (LL-TLAS) or Analytical Tunable Laser Absorption Spectroscopy (ATLAS) [1]. In the LL-TLAS mode, the emission is ideally matched with the central wavelength of the absorption line, thereby the overall laser intensity observed by the detector will be affected as the gas concentration is varied [1,2,3]. In the ATLAS mode, the laser line emission is scanned over the spectral region where the molecule absorbs [4,5,6,7,8,9]. Furthermore, this scanning task can be carried out by two different techniques: Direct Absorption (DA-ATLAS) and Wavelength Modulation (WM-ATLAS) [4]. In the DA-ATLAS case, the laser wavelength is tuned over the spectral region where the gas absorption line occurs; therefore, based on the Beer–Lambert law, the transition line shape and its integrated absorbance can be determined straightforwardly [5]. Here, the laser wavelength is driven typically with a saw-tooth or triangular waveform of a certain frequency. In the WM-ATLAS case, the laser wavelength is also driven with a typical saw-tooth or triangular waveform, but this is also modulated with another faster signal waveform [4,5]. Usually, sensors based on the WM-ATLAS technique have a minimum detectable absorbance in the order of 10${}^{-6}$; this is quite superior to that typically achieved by sensors based on DA-ATLAS, which is in the order of 10${}^{-3}$ [10]. For instance, the sensor based on WM-ATLAS designed by [6] was able to simultaneously detect CH${}_{4}$ and H${}_{2}$S with a minimum detection limit of 1.1 ppm m and 15 ppm m, respectively. An example of a typical sensor based on DA-ATLAS is the one presented by [7], which can detect a minimum concentration of 0.2% of CH${}_{4}$. One disadvantage of WM-ATLAS sensors is that their highest detectable concentration typically lies within a narrow concentration range (<1%), while sensors based on DA-ATLAS usually have a larger dynamic range [10]. Furthermore, as in the ATLAS sensors, the laser line emission is tuned, therefore these will generate an output signal in the time domain. From this signal output, different spectroscopic parameters can be determined depending on the sensor configuration. For instance, there are algorithms in which the measured sensor signals are fitted with Voigt [5,8,11], Lorentzian [9], Voigt-Lorentzian or Voigt-Gaussian functions [4] in order to determine one or more spectroscopic parameters such as the line strength, the line position and the lower-state energy of one or multiple absorption lines [11]. However, for some applications, it is only necessary to determine the gas concentration at ambient temperature and at atmospheric pressure. For instance, for coal mine hazard detection applications, CH${}_{4}$ sensors with a dynamic range from 0–100% are of interest [12,13], as well as, for this application, sensors to monitor CO, O${}_{2}$, C${}_{2}$H${}_{2}$ and CO${}_{2}$ are required [12]. Here, for gas concentration sensors, simple algorithms can be applied to determine the concentration from the output signal. For example, a relationship can be established between the concentration and one statistics feature of the signal generated by the sensor. Some commonly-used statistics features are the peak amplitude [14], the area under the curve [10] and the Root-Mean Square (RMS) [7]. These kinds of relationships are easy to compute, and therefore, the gas concentration can be determined straightforwardly. However, sensors based on these algorithms commonly have a limited dynamic range since most of these statistics features (peak amplitude, area and RMS) will linearly increase with the concentration up to a certain level. Afterwards, the statistics feature will increase slowly with the concentration due to the effect of saturation in the gas cell. In this range, the relationship between the statistics feature and the concentration will have a nonlinear behavior. Consequently, the sensitivity to the gas concentration will have a quasi constant trend for a limited dynamic range, and afterwards, it will drops rapidly with the concentration. Different optimized designs have been proposed to widen the dynamic range while keeping a high sensitivity. For instance, the sensor developed by [10] reached a dynamic range of 0.005–50% CH${}_{4}$, and in this particular case, the concentration was determined by means of the relationship between the area under the curve and the concentration. Hence, there are several gas sensors based on the DA-ATLAS technique for which designers have improved the minimum detectable absorption and also have increased the sensitivity over a certain dynamic range. Some of these enhancements have been based on improving the optical arrangements, optimizing the optical component characteristics and the electronic stages used to recover and to process the signals.

In this paper, a tailored algorithm to widen the dynamic range of sensors based on DA-ATLAS, is presented. Moreover, based on the proposed algorithm, it is shown that it is possible to establish a quasi linear relationship between a tailored statistics feature and the concentration within the wider dynamic range. This consequently allowed us to get a high and quasi constant sensitivity within this concentration range. Furthermore, this algorithm can be applied to currently-designed sensors since it is only necessary to change the way in which the sensor signal output is processed. This new algorithm is based on the combination of different statistical features of the sensor signal output instead to consider just one statistics feature (area, peak amplitude, RMS). This type of statistical analysis is commonly used in digital image processing for texture measurements [15,16,17,18,19,20]. Finally, in order to support the general performance of the algorithm, some experimental results are presented.

## 2. Basic Principles of DA-ATLAS Gas Sensors

The principle of DA-ATLAS sensing is based on the amount of light absorbed when it passes through a gas sample. Hence, it is necessary to measure the absorption per each wavelength. Therefore, the laser is tuned over a certain spectral range to resolve the absorption line. The transmitted light intensity through a gas cell can be described by the Beer–Lambert law, which is given as:

(1) $$I\left(\lambda ,C,l\right)=I_{0}\left(\lambda \right)\exp\left(-Cl\alpha \left(\lambda \right)\right),$$

where $I_{0}\left(\lambda \right)$ is the laser intensity before the gas cell, *C* is the gas concentration, *l* is the gas cell pathlength and $\alpha \left(\lambda \right)$ is the monochromatic absorption coefficient. The latter was calculated by using the parameter of the HITRAN spectroscopy database [21]. Moreover, the proportion of light absorbed by the gas sample through the cell can be expressed by:

(2) $$a\left(\lambda ,C,l\right)=1-\frac{I\left(\lambda ,C,l\right)}{I_{0}\left(\lambda \right)}=1-\exp\left(-Cl\alpha \left(\lambda \right)\right).$$

**Figure 1:**
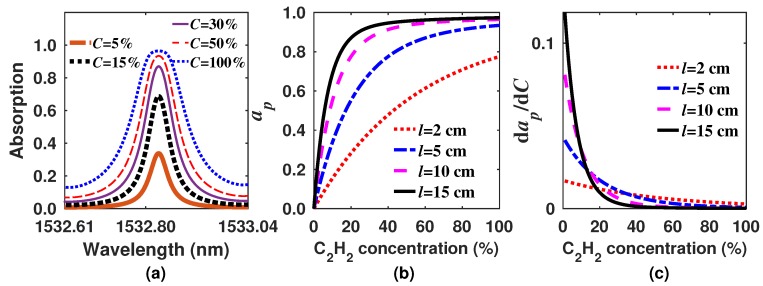
(**a**) Simulated C${}_{2}$H${}_{2}$ absorption spectra considering a pathlength of 10 cm and different concentrations; here, we considered that the laser is scanned over the absorption line; (**b**) peak absorption as a function of the concentration considering different pathlengths; (**c**) sensitivity of the absorption peaks as a function of the C${}_{2}$H${}_{2}$ concentration.

Simulated absorption profiles of $a\left(C,l,\lambda \right)$ for different concentration levels and considering that the laser is scanned from 1532.61–1533.04 nm, where one ro-vibrational line of C${}_{2}$H${}_{2}$ occurs, are shown in Figure 1a. Here, it can be observed that the peak amplitude $a_{p}=\max\left(a\left(C,l,\lambda \right)\right)$ rapidly increases with the concentration. However, how fast the $a_{p}$ reaches the maximum value will depend not only on the sample concentration, but also on the gas cell pathlength. For instance, in Figure 1b, the peak amplitude of the absorption for different concentrations and cell lengths is shown. Here, it can be observed that the longer the cell pathlength, the more rapidly the signal peak amplitude will reach one. Besides, as it approaches one, it becomes almost flat for the rest of the concentration range. Consequently, if the concentration is determined based on the peak amplitude, it is possible to optimize the sensor by selecting the gas cell pathlength in order to have either a low and quasi constant sensitivity over the wide concentration region or have a quite large sensitivity for a narrow range of low concentration levels, since after a certain concentration level, the sensitivity will tend to zero (Figure 1c). In this case, the sensitivity was calculated as $da_{p}/dC$. Hence, it can be useful to have a different option to analyze and process these absorption profiles, which are the sensor output, to determine the concentration with higher precision, especially in the region where the sensitivity is close to zero. Here, we propose a new algorithm, which helps to conserve the high sensitivity over a much wider dynamic range and also provides a quasi constant sensitivity behavior within this wider range. Additionally, it allows us to have a quasi constant sensitivity over the wide measurement range.

### Basic DA-ATLAS Gas Sensor with Two Optical Channels

**Figure 2:**
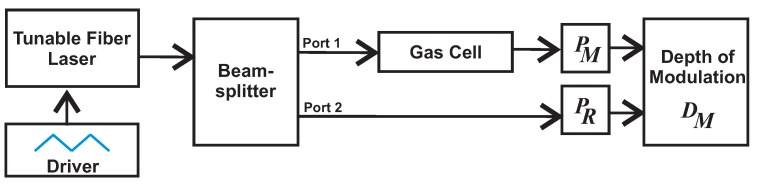
Block diagram of a Direct Absorption Tunable Laser Absorption Spectroscopy (DA-ATLAS) sensor with two optical channels.

There exist different sensor configurations to perform the sensing based on DA-ATLAS mode. One of these configurations, which is very popular, is that based on dual channels [3,4,5,7,22]. In this configuration, two channels are ideally formed by equally splitting the light beam before it enters the gas cell. In the measurement channel, the beam passes through the gas cell, and at the output, it is recorded by one optical detector. In the reference channel, the beam does not pass by the measurement cell, and therefore, it directly arrives at another detector. In Figure 2 is shown a general block diagram of a dual channel sensing setup. One advantage of this configuration is that the signals of the measurement and the reference channels can be either subtracted, divided or mathematically combined in order to calculate the transmission, the visibility or the depth of modulation (DM). The $D_{M}$ is an interesting measurement since it is robust to fluctuations of the light source [23,24], and it can be described as:

(3) $$\begin{array}{cc} D_{M}\left(C,l,\lambda \left(t\right)\right) & =\frac{P_{R}\left(\lambda \left(t\right)\right)-gP_{M}\left(C,l,\lambda \left(t\right)\right)}{P_{R}\left(\lambda \left(t\right)\right)+gP_{M}\left(C,l,\lambda \left(t\right)\right)}, \end{array}$$

where $P_{R}\left(\lambda \left(t\right)\right)$ and $P_{M}\left(C,l,\lambda \left(t\right)\right)$ are the power registered by the reference and the measurement detectors respectively when the laser wavelength is at position $\lambda \left(t\right)$. Here, as the laser wavelength will be scanned periodically, therefore it can be represented as a function of time $\left(t\right)$. Moreover, $D_{M}$ is unitless, and in our case, *g* is a proportionality constant to take into account losses or an unbalanced beam splitting ratio, ideally g=1. In a practical way, *g* can be calculated as the ratio between the signals provided by the reference and the measurement detectors when the target gas concentration is 0%. Furthermore, Equation (3) demonstrates the advantage of using the two-channel setup, since any source intensity fluctuation will affect both channels with the same magnitude, and therefore, their effects will be minimized when the $D_{M}$ is computed.

## 3. Tailoring an Algorithm for Enhancement of the Sensor Sensitivity to the Gas Concentration

**Figure 3:**
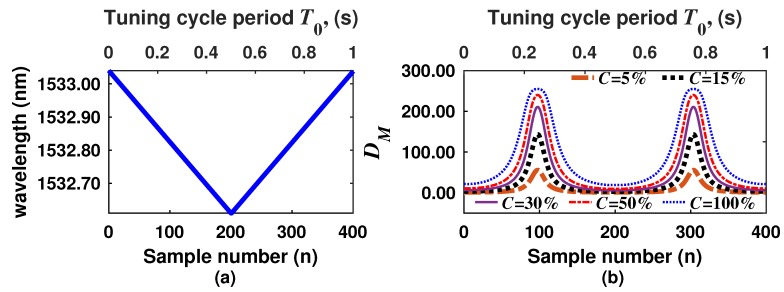
(**a**) Laser wavelength as a function of time; (**b**) depth of modulation as a function of time, considering different concentrations of C${}_{2}$H${}_{2}$ and l=10 cm.

In the DA-ATLAS technique, the laser is tuned in one direction to scan the spectral region where an absorption line of the target molecule occurs, and afterwards, it is returned to its initial position (Figure 3a). This spectral scan can be driven by using, for instance, a saw tooth or a triangular waveform. As an example and in order to explain our algorithm methodology, let us consider that a C${}_{2}$H${}_{2}$ sensor is going to be designed, targeting the absorption line occurring at 1532.8302 nm with a 10-cm gas cell pathlength. Moreover, let us to consider that the laser is tuned within the 1532.61–1533.04 nm range and it is driven with a triangular waveform (Figure 3a). Here, the time duration of the tuning cycle period $\left(T_{0}\right)$ will depend on the frequency at which the laser is tuned. Hence, as the laser is periodically scanned over time, therefore the $D_{M}$ of the output will have a particular waveform in the time domain, as is shown in Figure 3b. For this figure, different concentrations of C${}_{2}$H${}_{2}$ were considered, and it can be appreciated how the peak amplitude of the signal increases with the gas concentration. Furthermore, the $D_{M}$ signal was normalized to 255 considering the standard gray tone scale of image processing. From this figure, it also can be observed that different statistical features of the sensor signal output, such as the peak to peak amplitude, the mean, the RMS and the DC level, are affected by the concentration. Therefore, if the gas concentration is determined based only on the peak amplitude of the signal, more valuable information is wasted. Thus, it is possible to build new algorithms to extract additional information of the statistics features of the sensor output waveforms in order to enhance some sensor characteristics such as the sensitivity and the gas concentration dynamic range.

**Figure 4:**
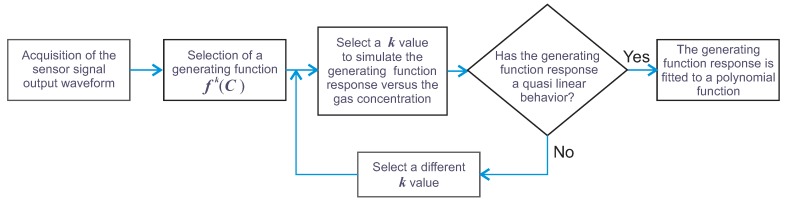
Flow diagram of the proposed algorithm.

The algorithm proposed in this work is based on the analysis of the sensor signal output waveforms. Here, for simplicity and normalization purposes, we will consider the number of samples (n) forming a laser tuning cycle. In this way, *N* will be the total number of samples composing each tuning cycle period $\left(T_{0}\right)$, and it also represents the number of scanning steps considered to complete one laser tuning cycle (Figure 3). The general flow diagram of the algorithm proposed in this work is presented in Figure 4. This steps should be performed for designing a particular gas sensor.

According to the flow diagram for implementing the new algorithm, it is necessary to build our generating functions $\left(f^{k}\left(C\right)\right)$, which are based on some statistical features of the sensor signal output, such as the mean ($m^{1}$), the peak amplitude of the signal (*A*), the entropy (*h*), the standard deviation (σ) and the *k*-th momentum over the origin ($m^{k}$). These statistics features can be expressed by the following Equations:

(4) $$m^{k}\left(C\right)=\sum_{j=1}^{N}\frac{{\left[D_{M}\left(C,j,\right)\right]}^{k}}{N},$$

(5) $$\sigma \left(C\right)=\sum_{j=1}^{N}\frac{{\left[D_{M}\left(C,j\right)-m^{1}\left(C\right)\right]}^{2}}{N},$$

(6) $$h\left(C\right)=\sum_{j=1}^{N}\frac{D_{M}\left(C,j\right)\ln\left[DM\left(C,j\right)+1\right]}{N},$$

(7) $$A\left(C\right)=\max\left(D_{M}\left(C,j\right)\right).$$

**Figure 5:**
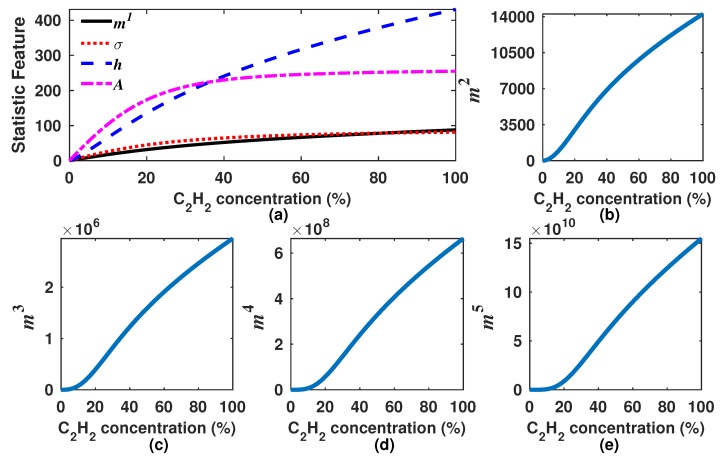
Statistics features of the sensor output waveforms as a function of C${}_{2}$H${}_{2}$. (**a**) the mean ($m^{1}$), the standard deviation (σ), the entropy (*h*) and the peak amplitude (*A*) of $D_{M}$ are shown; (**b–e**) some *k*-th moments over the origin ($m^{k}$) of $D_{M}$ are presented.

In Figure 5, the *A*, σ, *h* and $m^{1}$–$m^{5}$ features from $D_{M}$ waveforms as a function of the C${}_{2}$H${}_{2}$ concentration are shown. From Figure 5a, it can be observed that the parameter *A* is the one that most rapidly increases with low concentration levels (0–20%). However for higher concentration levels (20–100%), *A* becomes quite flat meaning that it has become insensitive to concentration changes. Moreover, it can be appreciated that for higher concentration levels, the most sensitive statistics feature is the entropy (*h*). Furthermore, it also can be appreciated that the mean and the σ present a lower sensitivity to the gas concentration. Finally, it can be appreciated in Figure 5b–e that *k*-th moments over the origin are quite sensitive for higher concentrations; however, these have the disadvantage that for low concentration levels, their response is close to zero. For instance, in Figure 5e, it can be observed that below a 20% C${}_{2}$H${}_{2}$ concentration, $m^{5}$ is practically zero and after this level presents a high slope for the range from 20–100%. Therefore, it can be concluded from this first analysis that some statistics features are quite sensitive to low concentrations levels (*A* and *h*) and others to high levels ($m^{k}$).

### 3.1. Generating Functions

**Figure 6:**
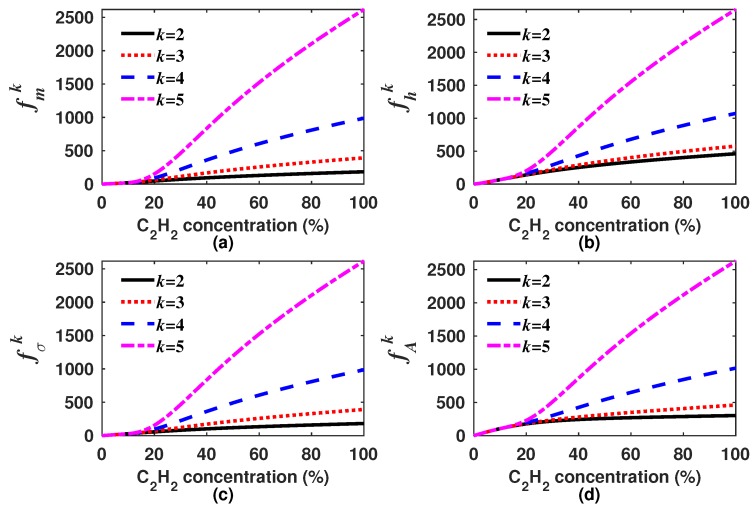
Generating functions (**a**) $f_{m}^{k}$; (**b**) $f_{h}^{k}$; (**c**) $f_{\sigma }^{k}$; and (**d**) $f_{A}^{k}$ versus the C${}_{2}$H${}_{2}$ concentration. In all cases we considered *k* as 2, 3, 4 and 5.

In order to take advantage of the fact that each statistics feature is more sensitive to different concentration intervals, different ways to combine the *k*-th moment (k>1), *A*, σ, *h* and $m^{1}$ were analyzed. In this way several new functions can be built. From these new functions, we selected one set, called here generating functions, which can be expressed as:

(8) $$f_{m}^{k}=\sqrt{{\left(m^{1}\right)}^{2}+{\left(\frac{m^{k}}{w^{k-1}}\right)}^{2}},$$

(9) $$f_{h}^{k}=\sqrt{h^{2}+{\left(\frac{m^{k}}{w^{k-1}}\right)}^{2}},$$

(10) $$f_{\sigma }^{k}=\sqrt{\sigma^{2}+{\left(\frac{m^{k}}{w^{k-1}}\right)}^{2}},$$

(11) $$f_{A}^{k}=\sqrt{A^{2}+{\left(\frac{m^{k}}{w^{k-1}}\right)}^{2}},$$

where *w* is a weighting constant to scale the magnitude of the high order moments. In our case, *w* was considered as the maximum of $m^{1}$ within the measurement range, which corresponds to the C${}_{2}$H${}_{2}$ concentration of 100% ($w=m^{1}\left(100\%\right)$). In Figure 6, it can be observed that the slope of all the generating functions becomes larger the higher the order of the moment about the origin is. Moreover, it also can be appreciated that these functions will have a well-defined inflection point occurring when the *k*-th moment starts to govern the behavior of the generating function.

### 3.2. Selection of the Optimum Generating Function

**Figure 7:**
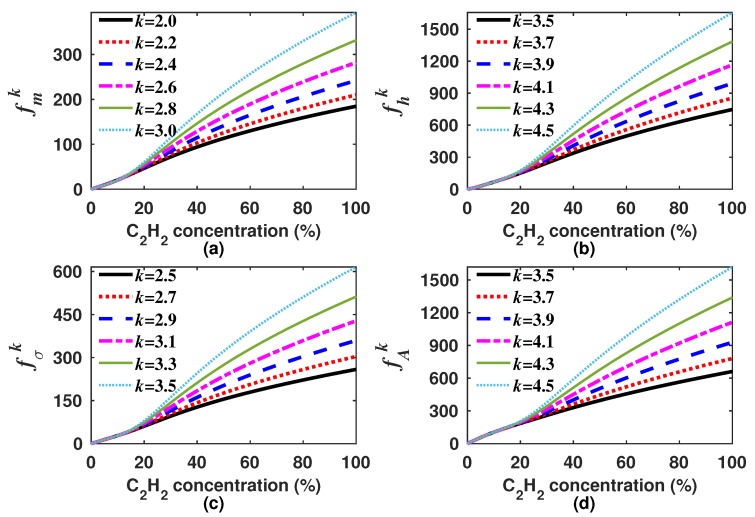
Generating functions (**a**) $f_{m}^{k}$; (**b**) $f_{h}^{k}$; (**c**) $f_{\sigma }^{k}$; and (**d**) $f_{A}^{k}$ versus the C${}_{2}$H${}_{2}$ concentration for an interval of *k* values that generate functions with a quasi-linear behavior.

**Figure 8:**
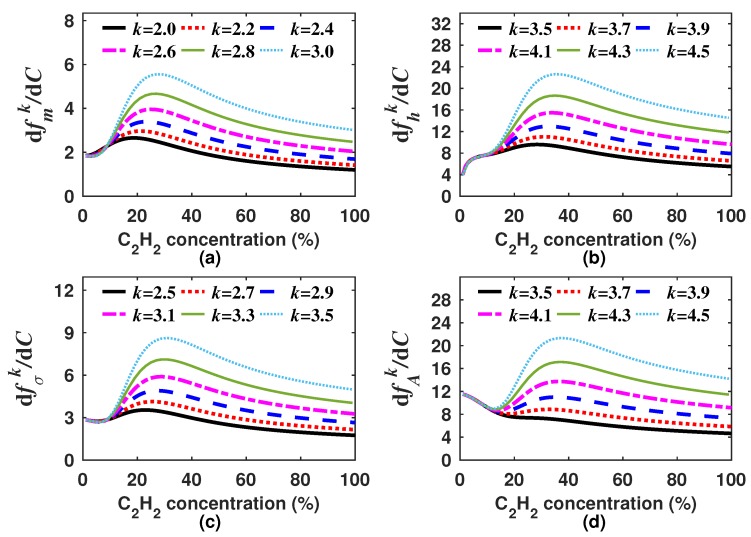
Sensitivities of the generating functions (**a**) $f_{m}^{k}$; (**b**) $f_{h}^{k}$; (**c**) $f_{\sigma }^{k}$ and (**d**) $f_{A}^{k}$ versus the C${}_{2}$H${}_{2}$ concentration for an interval of *k* values that generate functions with a quasi linear behavior

**Figure 9:**
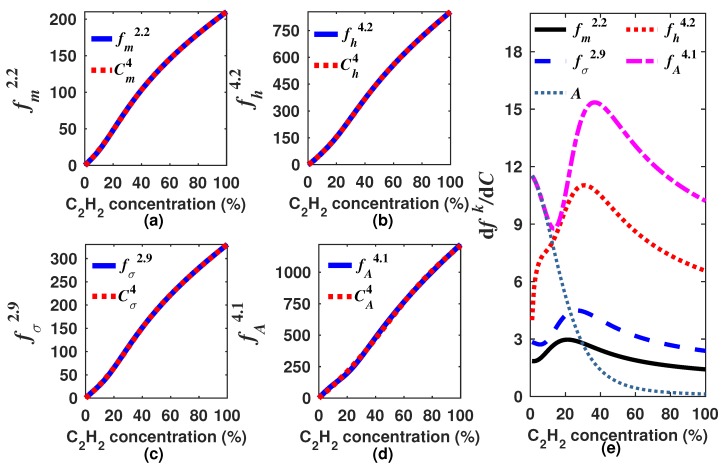
Simulated optimal generating functions (**a**) $f_{m}^{2.2}$; (**b**) $f_{h}^{4.2}$; (**c**) $f_{\sigma }^{2.9}$; and (**d**) $f_{A}^{4.1}$ and their corresponding polynomial fits; (**e**) C${}_{2}$H${}_{2}$ sensitivities of each one of the optimal generating functions and of that obtained when only the peak amplitude of $D_{M}$ is considered.

As there are infinite possible *k* values, it is necessary to select only one for each generating function (Equations (8)–(11)). In our case, we decided to look for the *k* values for which the built generating functions have a quasi linear response to the concentration. Moreover, in general terms, the sensitivity can be calculated as the derivative of the generating function with respect to the gas concentration $\left(df^{k}/dC\right)$. Since we are looking for functions with quasi linear response to the concentration (Figure 7), therefore the sensitivity must have a quasi constant behavior within the measurement range. It is important to point out that the *k* value will not necessarily be the same for each generating function. For instance, for our design example, we found that the optimal *k* was within the range of 2.0–3.0, 3.5–4.5, 2.5–3.5 and 3.5–4.5 for $f_{m}^{k}$, $f_{h}^{k}$, $f_{\sigma }^{k}$ and $f_{A}^{k}$, respectively. In Figure 7 and Figure 8, the generating functions ($f^{k}$) for these *k* intervals, in steps of 0.2, and their corresponding sensitivities are shown, respectively. From Figure 8, it can be observed that the sensitivity of the four generating functions is improved with these *k* values since these are now much larger than zero and have a quasi constant profile for high concentrations. This shows that by using these algorithms, it is possible to enhance the sensitivity for higher concentrations, which is not possible when the concentration is determined based only on either the peak amplitude, the mean, the RMS or the standard deviation. After we find a range of *k* values for which the generation functions have a quasi linear behavior, just one of these *k* values must be selected for each function. For our example, we determined as the optimum *k* value 2.2, 4.2, 2.9 and 4.1 for $f_{m}^{k}$, $f_{h}^{k}$, $f_{\sigma }^{k}$ and $f_{A}^{k}$, respectively. In Figure 9a–d, it can be appreciated that each optimal generating function has a very linear behavior compared to that obtained when the *A*, $m^{1}$, σ or *h* are computed (Figure 5a). Moreover, these functions present some inflection points due to the combination of different functions and can be precisely fitted with a polynomial.

In this form, the gas concentration can be calculated by using the expression $C_{f}^{r}=\sum_{n=0}^{r}p_{n}{\left(f^{k}\right)}^{n}$, where $p_{n}$ are the polynomial coefficients and *r* is the degree of the polynomial (Figure 9a–d). In a practical sensor design, the algorithm should be performed only one time to determine the generating function and its optimal *k* value. Afterwards, it is only necessary to calculate the generating function based on the sensor output waveform and from this result determine the gas concentration by using the polynomial coefficients. Additionally, the sensitivity for each one of the optimal generating functions is presented in Figure 9e. Here, it can be observed that all of them have an enhanced sensitivity for a higher concentration than obtained with the peak amplitude *A* of the sensor signal output ($D_{M}$). Moreover, from the sensitivity results, it can be observed that $f_{h}^{k}$ and $f_{A}^{k}$ have a larger sensitivity for all the measurement range (0–100%); however, for low concentrations, $f_{h}^{k}$ shows a lower sensitivity than that obtained with $f_{A}^{k}$. To the contrary, $f_{A}^{k}$ has a high sensitivity to low concentrations similar to that obtained when *A* is used, but due to the *k*-moment contribution, it keeps its high sensitivity for the full measurement range. This point demonstrates that the measurement range and the sensitivity of typical gas concentration sensors based on DA-ATLAS can be enhanced by using this kind of tailored algorithm.

## 4. Proof of Principle Gas Sensor Setup Based on DA-ATLAS

**Figure 10:**
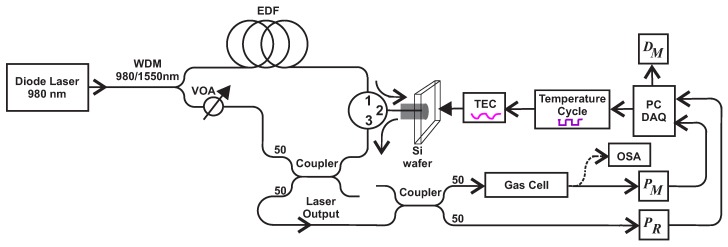
DA-ATLAS dual channel gas sensor setup. WDM, Wavelength Division Multiplexer; VOA, Variable Optical Attenuator; EDF, Erbium-Doped Fiber; TEC, Thermo Electric Cooler; OSA, Optical Spectrum Analyzer.

In order to test the viability of the tailored algorithms, particularly the expansion of the dynamic range and the enhancement of the sensitivity, a basic gas C${}_{2}$H${}_{2}$ sensor was implemented. It was based on a ring fiber tunable laser (Figure 10). Here, the light of a pigtailed diode laser emitting at λ = 980 nm and delivering a maximum output power of 300 mW was coupled to a Wavelength Division Multiplexer (WDM, Qphotonics QFBGLD-980-200) to pump an Erbium-Doped Fiber (EDF, Newport F-EDF-T3) of 3.4 m in length. Afterwards, the luminescence generated by the EDF traveled throughout the circulator (Thorlabs 6015-3) from Port 1 to Port 2 where a silicon wafer is placed. Port 3 of the circulator was spliced to a 50/50 coupler (Thorlabs 10202 A-50) in order to split the reflected interference spectrum of the wafer into two outputs. One of the 50% outputs was launched to a Variable Optical Attenuator (VOA), which was used to change the spectral gain of the laser to avoid laser emissions in the 1550-nm region. Finally, the VOA was spliced to the WDM-1550-nm port in order to close the ring cavity. The other 50% output of the reflected interference spectrum of the Si wafer was divided by a 50/50 fiber coupler, to form the measurement and the reference channels of the sensor. Here, the laser signal was monitored by using an Optical Spectrum Analyzer (OSA, Yokogawa AQ6370C) with a resolution of 0.02 nm. In this arrangement, a 4-nm laser tuning range without mode hopping was achieved by varying the wafer temperature with the Thermo Electric Cooler (TEC). Here, in order to have repetitive tuning cycles, the laser line wavelength was tuned over time by driving the TEC with a Proportional-Integral-Derivative (PID) controller, which was implemented with LabVIEW software (PC DAQ). In this laser setup, a bulk silicon wafer of 85 μm in thickness was used as a spectral selective filter [25]. Hence, the wafer acts as a Fabry–Perot Interferometer (FPI), and therefore, its reflection pattern can be shifted by varying the refractive index of silicon. This can be achieved by taking advantage of the thermo-optical properties of silicon [25]. Consequently, this allowed us to tune the laser emission wavelength to scan one ro-vibrational absorption line of the target molecule. In our case, we tuned the laser emission over the ro-vibrational line of C${}_{2}$H${}_{2}$ occurring at 1532.8302 nm at atmospheric pressure [21]. For the experiments carried out in this work, the VOA remained fixed. Here, the light beam of the reference channel is monitored with the $P_{R}$ optical detector, while the measurement channel is monitored with the $P_{M}$ detector. These signals are recovered with a Data Acquisition System (DAQ) and processed with a Personal Computer (PC). This stage also calculates the $D_{M}$ signal, which is the main output signal of our sensor.

### 4.1. Characterization of the Laser Line Tuning and Simulation of the Sensor Output

**Figure 11:**
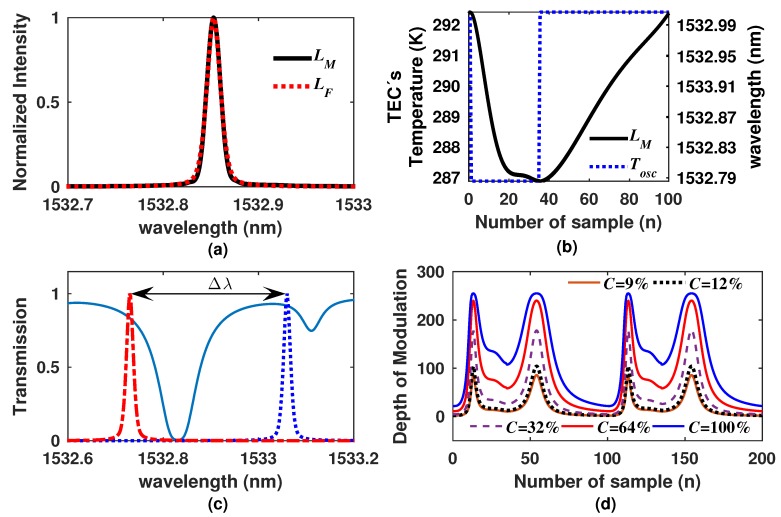
(**a**) Measured and fitted laser line emission; (**b**) laser wavelength as a function of the time as a consequence of the variation of the Si wafer temperature; (**c**) simulated transmission of C${}_{2}$H${}_{2}$ when C=50% and the initial and final positions of the laser emission tuning range; (**d**) simulated $D_{M}$ waveforms as a function of time for different C${}_{2}$H${}_{2}$ concentrations.

In order to be able to simulate the output waveform that will generate our gas sensor, we firstly characterized the laser line emission profile and its tuning behavior over time. The measured laser line profile $\left(L_{M}\right)$ is shown in Figure 11a. It was recorded with an optical spectrum analyzer (Yokowaga AQ6370C) with a resolution of 20 pm. This laser emission has a Full Width at Half Maximum (FWHM) of approximately 15 pm, and it was well fitted with a Gaussian Lorentzian Sum (GLS) function (Figure 11a), which is defined as [26]:

(12) $$L_{F}\left(\lambda \right)=\frac{1-q}{\left(\rho /2\right)\sqrt{2\pi }}\exp\left[-\frac{{\left(\lambda -\lambda_{0}\right)}^{2}}{2\rho^{2}}\right]+\frac{q}{\pi }\frac{\rho }{\rho^{2}+{\left(\lambda -\lambda_{0},\right)}^{2}},$$

where q=0.4 and ρ=FWMH/2. Moreover, by varying the TEC’s temperature from 287.9–292.0 K, it was possible to tune the laser emission from 1532.785–1533.000 nm $\left(\Delta \lambda =0.215\;\mathrm{nm}\right)$, as shown in (Figure 11b). Here, the laser line wavelength is scanned periodically over time. In our case, we developed a PID controller to drive the TEC’s voltage supply and to guarantee that the temperature remains over the wavelength scanning range. In this case, we did not optimize the PID in order to drive the TEC’s temperature with a linear behavior as when it is driven with sawtooth or triangular waveforms (Figure 3). In our TEC’s PID controller software program, we set the levels of temperature $\left(T_{osc}\right)$, and the laser emission was tuned following the function shown in Figure 11b. Moreover, by varying in this way the temperature of the Si wafer, the laser line was scanned almost completely over the ro-vibrational absorption line, as can be observed in Figure 11c. Finally, by using the simulated laser line emission and its tuning characterization, the $D_{M}$ for different concentrations was simulated, and the results are shown in Figure 11d; here, $D_{M}$ waveforms were normalized to 255.

### 4.2. Simulated Generating Functions for the Experimental Sensor Setup

**Figure 12:**
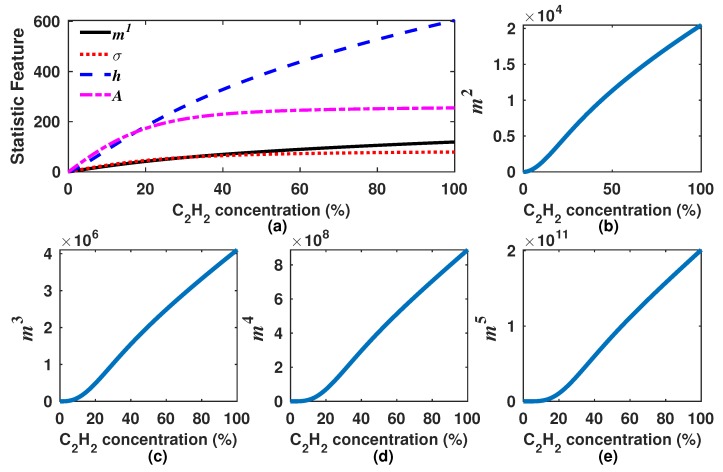
Simulated statistics features of our experimental gas sensor setup. (**a**) the mean ($m^{1}$), the standard deviation (σ), the entropy (*h*) and the peak amplitude (*A*) of $D_{M}$ are shown; (**b**–**e**) some *k*-th moments over the origin ($m^{k}$) of $D_{M}$ are presented

**Figure 13:**
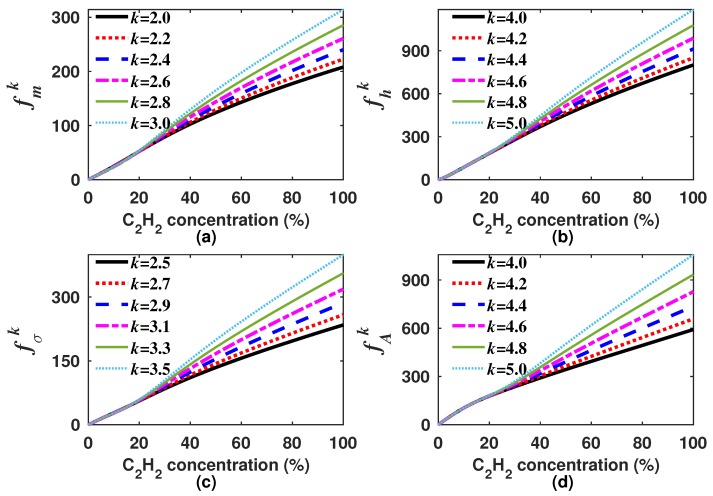
Simulated response of generating functions (**a**) $f_{m}^{k}$; (**b**) $f_{h}^{k}$; (**c**) $f_{\sigma }^{k}$; and (**d**) $f_{A}^{k}$ versus the C${}_{2}$H${}_{2}$ concentration for the experimental sensor setup. Here are shown only some intervals of *k* values for which generating functions can have a quasi linear behavior.

**Figure 14:**
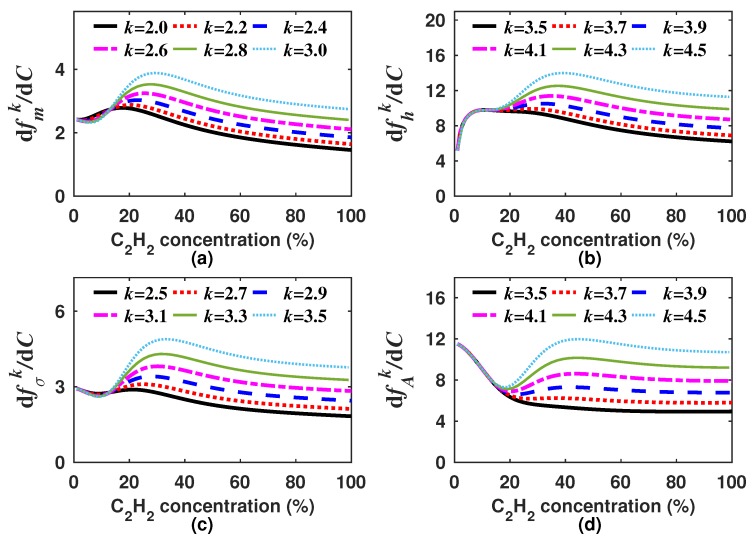
Sensitivity of the generating functions (**a**) $f_{m}^{k}$; (**b**) $f_{h}^{k}$; (**c**) $f_{\sigma }^{k}$ and (**d**) $f_{A}^{k}$ versus the C${}_{2}$H${}_{2}$ concentration that are shown in Figure 13. Here it shown an interval of *k* values that generate a quasi constant sensitivity.

**Figure 15:**
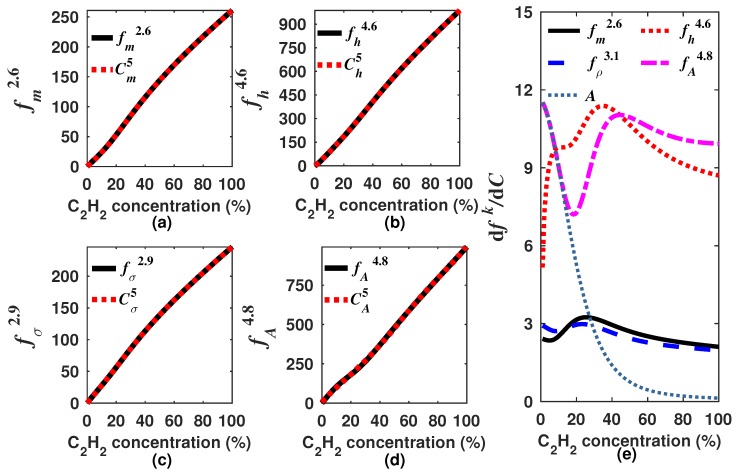
Simulated optimal generating functions (**a**) $f_{m}^{k}$; (**b**) $f_{h}^{k}$; (**c**) $f_{\sigma }^{k}$ and (**d**) $f_{A}^{k}$ for our experimental setup as a function of the C${}_{2}$H${}_{2}$ concentration; (**e**) simulated sensitivity of the optimal $f^{k}$ functions.

Once the $D_{M}$ signal was simulated, we proceeded to calculate the generating functions and to look for the optimal *k* value that provided the most constant sensitivity for each one. Firstly, the $m^{k}$, *h*, σ and *A* statistics features were calculated (Figure 12). Afterwards, based on Equations (8)–(11), the generating functions were calculated for some *k* values for which these functions provide the most constant sensitivity over a wider dynamic range. In Figure 13, some examples of functions obtained with some particular values of *k* are shown. Moreover, the sensitivity that can be achieved by using these functions is shown in Figure 14. Afterwards, we proceeded to select one *k* value for each generating function of this experimental case, which were 2.6, 4.6, 2.6 and 4.8 for $f_{m}^{k}$, $f_{h}^{k}$, $f_{\sigma }^{k}$ and $f_{A}^{k}$, respectively (Figure 15). Moreover, their sensitivities are shown in Figure 14e. From this figure, it can be observed that $f_{h}^{k}$ and $f_{A}^{k}$ have a greater sensitivity for high C${}_{2}$H${}_{2}$ concentrations and also that the $f_{A}^{k}$ function has a greater sensitivity for low concentration levels. Therefore, we considered that $f_{A}^{4.8}$ is the best option to be used for this gas sensor design since it has a larger concentration over the full concentration range. Finally, these optimal generating functions were fitted with a polynomial of the form $C_{f}^{5}=\sum_{n=0}^{5}p_{n}{\left(f^{k}\right)}^{n}$ to determine the concentration, and the $p_{n}$ constants are listed in Table 1.

### 4.3. Experimental Measurements

**Figure 16:**
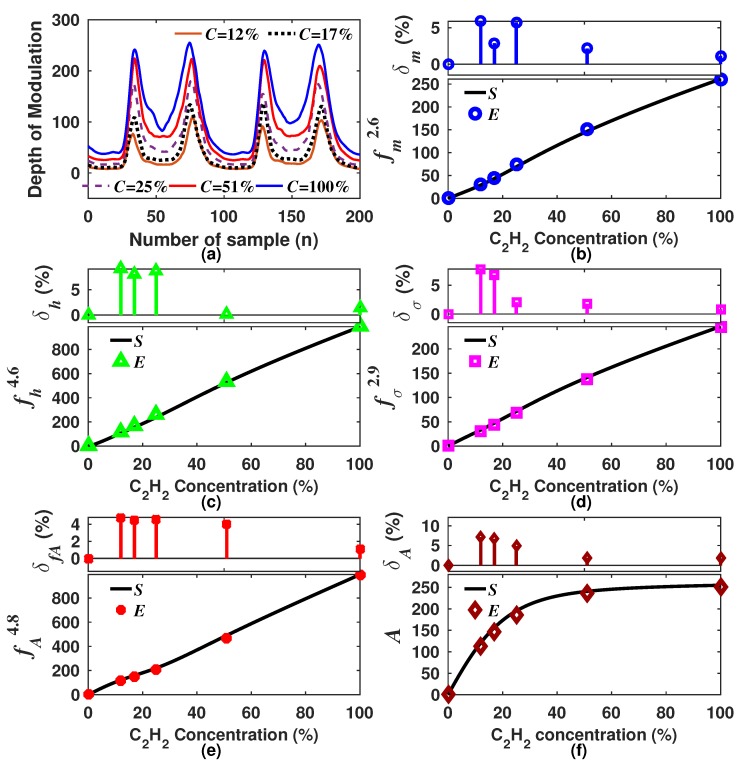
(**a**) Measured sensor output waveforms for different C${}_{2}$H${}_{2}$ concentrations; (**b**–**f**) simulated (*S*) and calculated statistics features obtained from experimental waveforms (*E*).

Now, in order to support our simulated results, we performed some measurements with different C${}_{2}$H${}_{2}$ concentrations. The measured $D_{M}$ waveforms for each concentration are shown in Figure 16a (see Table 1). Afterwards, for each one of these $D_{M}$ signals, the optimal generating functions $f_{m}^{2.6}$, $f_{h}^{4.6}$, $f_{\sigma }^{2.9}$ and $f_{A}^{4.8}$ were calculated.

Additionally, as an example of the typical results expected when the concentration is determined based on a single statistics feature, the peak amplitude of the $D_{M}$ waveforms was calculated. These results are shown in Figure 16b–e where it can be observed that all have the same tendency as the simulated results. In each graph is shown the error between the measured and the simulated results. It can be expressed as $\delta =|f_{E}^{k}-f_{S}^{k})/f_{S}^{k}|\times 100\%$. Here, let us point out that we consider that these errors are mainly due to systematic causes, and therefore, these can be reduced by further optimizing the sensing arrangement stages. In practice, after these generating function results are obtained, the C${}_{2}$H${}_{2}$ concentration can be determined by means of the polynomial coefficients listed in Table 1.

### 4.4. Experimental Sensitivity

**Figure 17:**
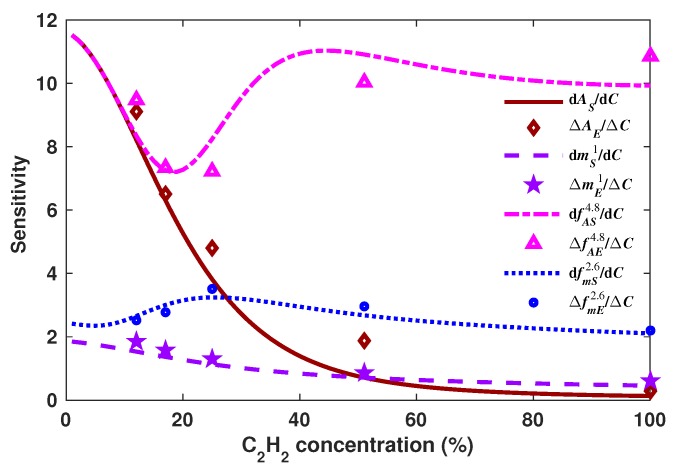
Simulated (*S*) and experimental (E) sensitivities for the generating functions $f_{A}^{4.8}$, $f_{m}^{2.6}$ and for the peak amplitude (*A*) and the mean ($m^{1}$).

One of the main points of the algorithm is to enhance the sensitivity over a larger dynamic range. As an example, the sensitivity obtained based on a single statistics feature (peak amplitude and mean) and that obtained with some generating functions ($f_{A}^{4.8}$ and $f_{m}^{2.6}$) are shown in Figure 17. For this case, the approximate sensitivity values for the experimental measurements *E* were calculated by using the relationship $\Delta f/\Delta \left(C_{s}\right)=\left[f\left(C_{s}\right)-f\left(C_{s-1}\right)\right]/\left(C_{s}-C_{s-1}\right)$. From Figure 17, it can be noted that the tailored functions provide a better sensitivity level over a much wider dynamic range. For instance, $f_{A}^{4.8}$ has a similar sensitivity to that of *A* within the concentration range from 0–17%, but after this level, the sensitivity obtained with the generating function is considerably larger. Similar behavior is observed when the sensitivity is obtained with the $f_{m}^{2.6}$ and $m^{1}$ functions. Thus, it can be concluded that generating functions can be suitable to keep a high sensitivity over a larger dynamic range compared with that obtained when it is determined based on a single statistics feature, such as the peak amplitude, the mean, the area or the RMS. Furthermore, of our tailored functions, $f_{A}^{k}$ was the best option since it has the highest sensitivity for low concentration levels, and it is kept quasi constant for the full measurement range.

## 5. Conclusions

In this work, a tailored algorithm to enhance the sensitivity of sensors based on DA-TLAS was presented. Here, it was demonstrated that with this algorithm, it is possible to have a quasi constant sensitivity over a much wider dynamic range than obtained when the concentration is determined based on a single statistics feature such as the peak amplitude, the area, the mean or the RMS of the sensor output waveform. This is possible by taking into account other statistical features of the sensor output waveforms, which usually are wasted. Moreover, it was shown that different functions can be built by combining statistics features in order to tailor an optimal generating function to enhance the sensitivity for certain concentration ranges. Finally, the algorithm was tested for one experimental sensor setup, and the obtained measurements are in agreement with the our simulated results.

## Figures and Tables

**Table 1:** Polynomial coefficients of the optimal generating functions.

Generating Function	*k*	*p*5 $\times \;10^{-11}$	*p*4 $\times \;10^{-9}$	*p*3 $\times \;10^{-5}$	*p*2 $\times \;10^{-3}$	*p*1 $\times \;10^{-1}$	*p*0
$f_{m}^{k}$	2.6	7.3252	−65.3400	2.207	−3.013	4.8895	−0.2699
$f_{h}^{k}$	4.6	0.0001	−0.0539	0.014	−0.105	1.2107	0.3209
$f_{\sigma }^{k}$	2.9	−66.7622	169.8169	−1.542	0.282	3.6539	−0.1325
$f_{A}^{k}$	4.8	−0.0330	0.8222	−0.0689	0.204	0.9397	−0.6466
